# Racial and ethnic disparities in esophageal cancer survival are greatest at curable stages: a population-based study

**DOI:** 10.1007/s10552-026-02194-5

**Published:** 2026-06-30

**Authors:** Saurabh Kalra, Cindy Medina Pabón, Deepak Kalra, WayWay M. Hlaing, Paulo S. Pinheiro

**Affiliations:** 1 Department of Public Health Sciences, University of Miami Miller School of Medicine, Miami, FL USA; 2 Department of Medicine, Sylvester Comprehensive Cancer Center, University of Miami Miller School of Medicine, Miami, FL USA; 3 Department of Neurology, Cleveland Clinic Florida, Weston, FL USA

**Keywords:** Esophageal cancer, Racial disparities, Survival disparities, Treatment disparities, Health equity, Population-based study

## Abstract

**Background:**

Racial and ethnic disparities in esophageal cancer outcomes are documented, but whether they differ by stage at diagnosis is unclear. Using population-based data from Florida, a diverse, Medicaid non-expansion state, we conducted a stage-stratified analysis of racial/ethnic differences in survival and treatment receipt.

**Methods:**

We analyzed 21,814 esophageal cancer cases from the Florida Cancer Data System (2005–2021). Using cause-specific Cox models, we estimated adjusted hazard ratios (aHRs) for cancer-specific mortality overall and by stage, adjusting for demographics, tumor characteristics, treatment, smoking, and socioeconomic factors. Multivariable logistic regression assessed racial/ethnic differences in receipt of surgery, chemotherapy, and radiation.

**Results:**

Patients were 81.0% non-Hispanic (NH) White, 8.0% NH Black, and 10.0% Hispanic. NH Black patients had higher overall mortality than NH White patients (aHR: 1.08; 95% CI 1.02–1.15). Stage-stratified models showed elevated mortality among NH Black patients at localized (aHR: 1.24; 95% CI 1.05–1.45) and regional stages (aHR: 1.19; 95% CI 1.07–1.35) compared with NH White patients, but lower mortality at distant stage (aHR: 0.88; 95% CI 0.80–0.98). NH Black patients had lower odds of surgery (aOR: 0.51; 95% CI 0.43–0.61) and chemotherapy (aOR: 0.79; 95% CI 0.70–0.90) compared with NH White patients; radiation receipt did not differ.

**Conclusion:**

Survival disparities were most pronounced at curable stages, indicating inequities in post-diagnosis management. Lower treatment receipt and  persistent survival differences after clinical adjustment suggest that structural and systemic factors contribute. These findings highlight a critical target for cancer control and underscore the need for system-level reforms to ensure equitable, stage-appropriate care.

**Supplementary Information:**

The online version contains supplementary material available at 10.1007/s10552-026-02194-5.

## Introduction

Esophageal cancer, including squamous cell carcinoma and adenocarcinoma, remains one of the most lethal malignancies in the United States, with persistently low survival, high recurrence rates, and marked racial and ethnic disparities [1]. National studies, primarily from the Surveillance, Epidemiology, and End Results (SEER) program and the National Cancer Database (NCDB), have reported worse outcomes among non-Hispanic (NH) Black patients compared with NH White patients [2–4]. However, Florida, the third most populous state, is not included in SEER, and NCDB data include only Commission on Cancer-accredited hospitals, limiting generalizability to the broader Florida population [5].

Florida’s status as a Medicaid non-expansion state and the Florida Cancer Data System’s (FCDS) routine capture of smoking status offer a unique opportunity to examine disparities in esophageal cancer outcomes [6–9]. FCDS is one of the few population-based registries with smoking information at diagnosis, allowing adjustment for smoking as an important prognostic factor for survival outcomes while assessing racial and ethnic disparities [10, 11].

Esophageal cancer treatment is strongly stage-dependent, with endoscopic resection or surgery central to localized disease (stages I–II), multimodality therapy for regional (locally advanced) disease (stages II–III), and palliative systemic therapy for distant disease (stage IV) [12–14]. These differences underscore the need to assess disparities within stage, rather than simply adjusting for stage, because early stages offer the greatest opportunity for curative treatment [15, 16]. Most prior studies have not examined how disparities vary across stages, potentially obscuring when inequities emerge and for whom they are most consequential.

Prior national analyses suggest survival gaps, particularly among NH Black esophageal squamous cell carcinoma patients experiencing substantially lower 5-year survival (~ 19%) compared to NH White patients (~ 28%) [3]. These differences may reflect under-treatment and socioeconomic barriers, rather than tumor biology [17, 18]. However, regional variation in healthcare access, insurance coverage, and demographic composition may limit the applicability of these findings to Florida, a uniquely diverse state with longstanding inequities in cancer care.

Using FCDS data, we examined whether racial and ethnic disparities in cancer-specific survival differ by stage at diagnosis and whether treatment disparities, including receipt of surgery and chemotherapy, help explain observed survival differences [8]. We also evaluated the prognostic impact of smoking status at diagnosis. We hypothesized that disparities in both treatment receipt and survival would be most pronounced at earlier stages, where effective and potentially curative therapies exist.

## Methods

### Data source and study population

We conducted a retrospective cohort study using data from the FCDS, a statewide population-based cancer registry that captures all incident cancer cases diagnosed in Florida [8]. The registry is certified by the North American Association of Central Cancer Registries (NAACCR) and maintains high standards for completeness and quality. This study included adults aged ≥ 18 years diagnosed with a first primary esophageal cancer (ICD-O-3 topography codes C15.0–C15.9) between 1 January 2005 and 31 December 2021. Histological subtypes were classified using ICD-O-3 morphology codes as adenocarcinoma, squamous cell carcinoma, and unspecified/other.

**Fig. 1:**
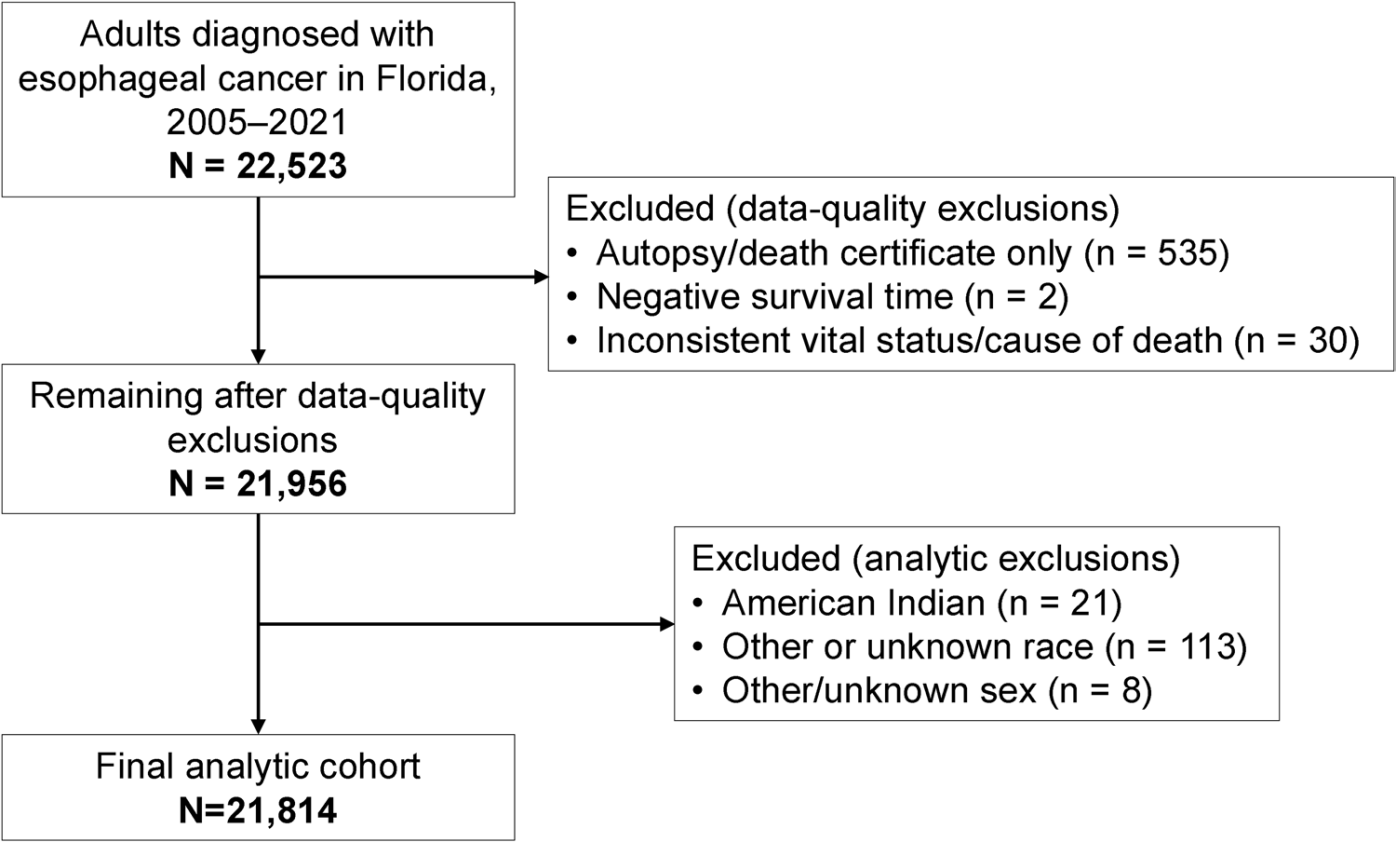
Flow diagram of cohort selection for esophageal cancer cases in the Florida Cancer Data System, 2005–2021

The final analytic sample included 21,814 patients after excluding cases with incomplete or inconsistent information, such as those diagnosed by autopsy or death certificate only (*n* = 535), with implausible survival times (*n* = 2), or inconsistent vital status and cause-of-death (*n* = 30) (Fig. 1). For analytic stability, we excluded patients with sex identified as “other” (*n* = 8) and those in small demographic groups, including American Indian (*n* = 21) and “Other” race (*n* = 113), due to limited representation.

### Variables

The primary outcome was esophageal cancer-specific mortality**,** defined as time in years from diagnosis to death or last follow-up. The all-patients model included individuals across all SEER Summary Stages, and separate stage-stratified models were estimated for localized, regional, and distant disease. Disease stage was classified using SEER Summary Stage 2000 (localized, regional, distant, unknown). Because stage is often conceptualized using the American Joint Committee on Cancer (AJCC) Tumor-Node-Metastasis (TNM) system, we note that SEER Summary Stage maps only approximately to TNM categories. Localized disease corresponds roughly to AJCC TNM stage I–IIA (with some stage IIB tumors also categorized as localized); regional disease includes most stage III tumors and a subset of stage IIB; and distant disease corresponds to metastatic (stage IV) tumors. Deaths not attributed to esophageal cancer were censored at the time of death. Patients alive on 31 December 2021 were censored on that date. Vital status and survival time were obtained by linking FCDS incidence records with Florida Bureau of Vital Statistics mortality data.

The primary exposure was race/ethnicity (mutually exclusive): NH White, NH Black, NH Asian, and Hispanic. Demographic covariates included sex (male/female), age at diagnosis (18–44, 45–54, 55–64, 65–74, 75–84, 85+), and marital status (married/partnered, separated/divorced/single, unknown). Socioeconomic variables included insurance type (private, Medicaid, Medicare, uninsured, other/unknown), and neighborhood poverty. Neighborhood-level poverty was defined using the census tract of residence and categorized into quartiles based on the percentage of residents living below the federal poverty level: 0 to  < 5%, 5 to  < 10%, 10 to  < 20%, and 20 to  < 100%.

Clinical factors included SEER Summary Stage at diagnosis (localized, regional, distant, unknown), tumor grade (well, moderate, poor, undifferentiated/unknown), and histologic subtype (as defined above). To reflect etiologic and prognostic differences by anatomic site and histology, we created a composite location–histology covariate with the following categories: upper (any histology; reference), middle (any histology), lower adenocarcinoma, lower non-adenocarcinoma and overlapping/other. This composite variable (upper as reference) was included in all multivariable models, along with tumor grade, given their established prognostic importance [19].

Upper esophageal tumors were selected as the reference group because they are relatively uncommon, predominantly squamous, and provide a clinically distinct comparator to middle and lower esophageal tumors, which are more adenocarcinomas associated with reflux and obesity. Squamous cell carcinomas, which predominantly arise in the upper and mid-esophagus, are strongly associated with tobacco and alcohol exposure. Accordingly, distinguishing lower esophageal adenocarcinomas from other histologic–anatomic subtypes and adjusting for smoking status at diagnosis enable a more precise assessment of underlying etiologic and prognostic differences [20]. Treatment variables included receipt of surgery (primary site), chemotherapy, and radiation (each coded as yes vs no/unknown) from structured treatment fields. Cases with missing data were retained and coded as “unknown” for the respective variable.

### Statistical analysis

**Fig. 2:**
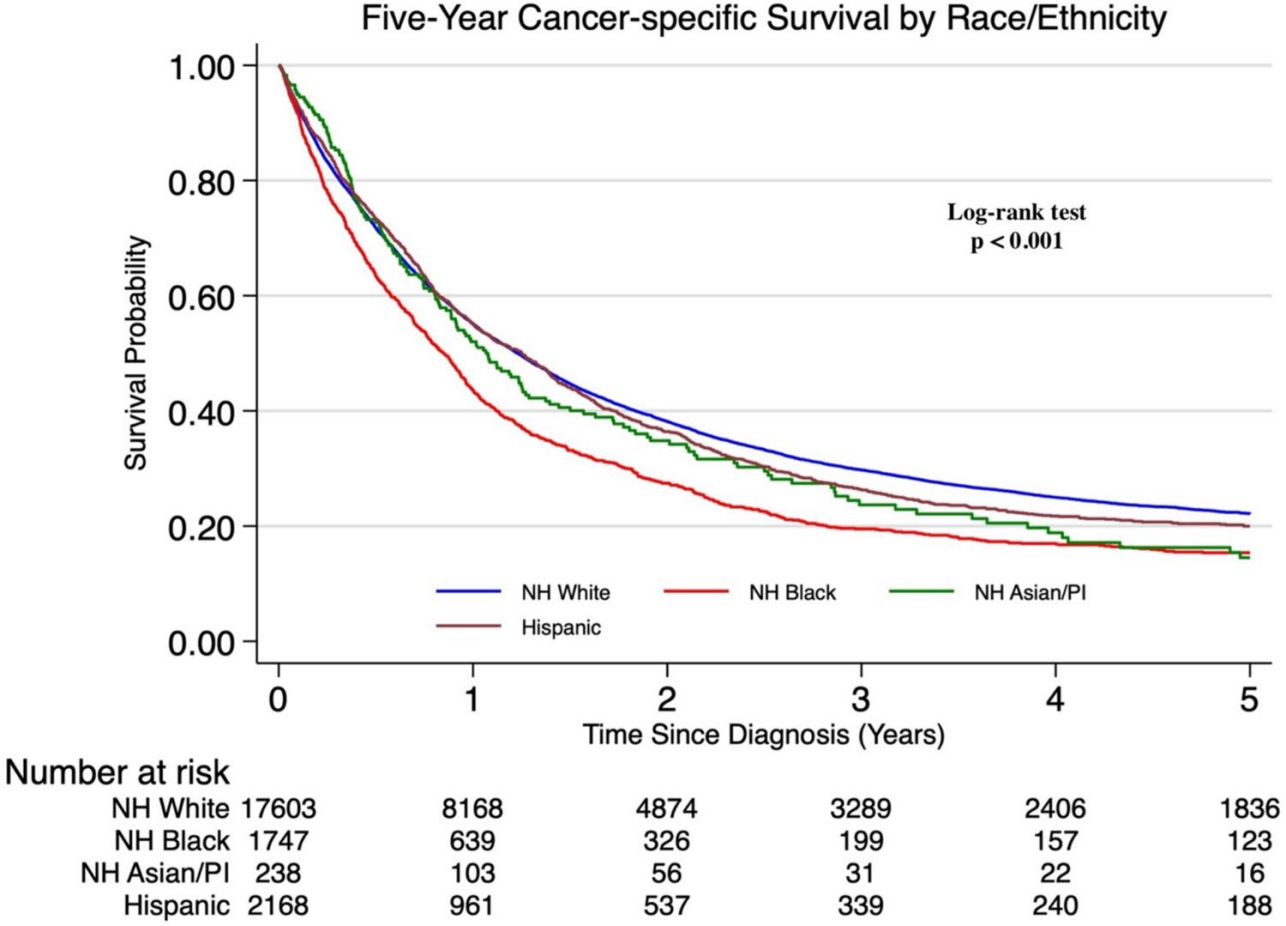
Five-Year Cancer-Specific Survival by Race/Ethnicity Among Patients Diagnosed with Esophageal Cancer, Florida Cancer Data System, 2005–2021. Kaplan–Meier cause-specific survival curves truncated at 5 years post-diagnosis. Survival probability is shown by race/ethnicity groups: Non-Hispanic (NH) White (blue), NH Black (red), NH Asian/PI (green), and Hispanic (maroon). Deaths not attributed to esophageal cancer were censored at the time of death. Log-rank test for survival differences across groups: *p* < 0.001

We summarized patient characteristics by race and ethnicity (Table 1) and conducted descriptive analyses stratified by stage at diagnosis (Supplementary Table 1S). Kaplan–Meier survival curves were generated to compare cancer-specific survival by race/ethnicity, with follow-up time truncated at 5 years (Fig. 2) [21].

**Table 1:** Characteristics of esophageal cancer patients by Race/ethnicity, Florida cancer data system, 2005–2021

Variable	Subcategory	All Patients (*n* = 21,814) *n* (%)	NH White (*n* = 17,655) *n* (%)	NH Black (*n* = 1,749) *n* (%)	NH Asian/Pacific Islander (*n* = 238) *n* (%)	Hispanic (*n* = 2,172) *n* (%)	*p*-value
Demographics							
Age, median (IQR), years		69 (61–77)	70 (62–77)	65 (58–74)	68 (59–75)	69 (60–77)	< 0.001
Age group	18–44	410 (1.9%)	268 (1.5%)	59 (3.4%)	11 (4.6%)	72 (3.3%)	< 0.001
	45–54	1,892 (8.7%)	1,429 (8.1%)	225 (12.9%)	22 (9.2%)	216 (9.9%)	
	55–64	5,159 (23.6%)	4,015 (22.7%)	567 (32.4%)	59 (24.8%)	518 (23.8%)	
	65–74	7,191 (33.0%)	5,922 (33.5%)	496 (28.4%)	84 (35.3%)	689 (31.7%)	
	75–84	5,383 (24.7%)	4,503 (25.5%)	328 (18.8%)	51 (21.4%)	501 (23.1%)	
	85 +	1,779 (8.2%)	1,518 (8.6%)	74 (4.2%)	11 (4.6%)	176 (8.1%)	
Sex	Male	17,092 (78.4%)	14,030 (79.5%)	1,181 (67.5%)	176 (73.9%)	1,705 (78.5%)	< 0.001
	Female	4,722 (21.6%)	3,625 (20.5%)	568 (32.5%)	62 (26.1%)	467 (21.5%)	
Marital status	Unmarried	3,675 (16.8%)	2567 (14.5%)	614 (35.1%)	38 (16.0%)	456 (21.0%)	< 0.001
	Married or living with a partner	12,022 (55.1%)	10,163 (57.6%)	596 (34.1%)	133 (55.9%)	1,130 (52.0%)	
	Separated/widowed	4,861 (22.3%)	3,926 (22.2%)	427 (24.4%)	52 (21.8%)	456 (21.0%)	
	Unknown	1,256 (5.8%)	999 (5.7%)	112 (6.4%)	15 (6.3%)	130 (6.0%)	
Insurance	Private	4,442 (20.4%)	3,593 (20.4%)	285 (16.3%)	62 (26.1%)	502 (23.1%)	< 0.001
	Medicaid	1,276 (5.8%)	754 (4.3%)	290 (16.6%)	22 (9.2%)	210 (9.7%)	
	Medicare	12,542 (57.5%)	10,519 (59.6%)	815 (46.6%)	108 (45.4%)	1,100 (50.6%)	
	Uninsured	766 (3.5%)	482 (2.7%)	127 (7.3%)	16 (6.7%)	141 (6.5%)	
	Other	2,788 (12.8%)	2,307 (13.1%)	232 (13.3%)	30 (12.6%)	219 (10.1%)	
Poverty level	Lowest	2,613 (12.0%)	2,328 (13.2%)	83 (4.7%)	35 (14.7%)	167 (7.7%)	< 0.001
	Low middle	6,520 (29.9%)	5,714 (32.4%)	216 (12.3%)	75 (31.5%)	515 (23.7%)	
	High middle	8,041 (36.9%)	6,624 (37.5%)	508 (29.0%)	92 (38.7%)	817 (37.6%)	
	Highest	4,461 (20.5%)	2,841 (16.1%)	925 (52.9%)	35 (14.7%)	660 (30.4%)	
	Non-response	179 (0.8%)	148 (0.8%)	17 (1.0%)	1 (0.4%)	13 (0.6%)	
Tumor characteristics							
Stage	Localized	4,160 (19.1%)	3,439 (19.5%)	304 (17.4%)	38 (16.0%)	379 (17.4%)	0.153
	Regional	6,370 (29.2%)	5,127 (29.0%)	515 (29.4%)	81 (34.0%)	647 (29.8%)	
	Distant	6,514 (29.9%)	5,237 (29.7%)	551 (31.5%)	68 (28.6%)	658 (30.3%)	
	Unknown	4,770 (21.9%)	3,852 (21.8%)	379 (21.7%)	51 (21.4%)	488 (22.5%)	
Grade	Well differentiated (grade 1)	797 (3.7%)	652 (3.7%)	64 (3.7%)	8 (3.4%)	73 (3.4%)	< 0.001
	Moderately differentiated (grade 2)	4,412 (20.2%)	3,499 (19.8%)	438 (25.0%)	50 (21.0%)	425 (19.6%)	
	Poorly differentiated (grade 3)	5,381 (24.7%)	4,422 (25.0%)	414 (23.7%)	51 (21.4%)	494 (22.7%)	
	Undifferentiated (grade 4)	159 (0.7%)	124 (0.7%)	12 (0.7%)	1 (0.4%)	22 (1.0%)	
	Unknown	11,065 (50.7%)	8,958 (50.7%)	821 (46.9%)	128 (53.8%)	1,158 (53.3%)	
Histology	Adenocarcinoma (EAC)	13,054 (59.8%)	11,472 (65.0%)	340 (19.4%)	89 (37.4%)	1,153 (53.1%)	< 0.001
	Squamous cell carcinoma	6,161 (28.2%)	4,045 (22.9%)	1,255 (71.8%)	128 (53.8%)	733 (33.7%)	
	Other	2,599 (11.9%)	2,138 (12.1%)	154 (8.8%)	21 (8.8%)	286 (13.2%)	
Tumor Location	Upper	1,621 (7.4%)	1,180 (6.7%)	226 (12.9%)	32 (13.5%)	183 (8.4%)	< 0.001
	Middle	3,298 (15.1%)	2,288 (13.0%)	575 (32.9%)	58 (24.4%)	377 (7.4%)	
	Lower EAC	10,005 (45.9%)	8,866 (50.2%)	229 (13.1%)	65 (27.3%)	845 (38.9%)	
	Lower non-EAC	2,832 (13.0%)	2,143 (12.1%)	361 (20.6%)	36 (15.1%)	292 (13.4%)	
	Overlapping/Other	4,058 (18.6%)	3,178 (18.0%)	358 (20.5%)	47 (19.8%)	475 (21.9%)	
Treatment characteristics							
Chemotherapy	No/unknown	9,384 (43.0%)	7,520 (42.6%)	822 (47.0%)	84 (35.3%)	958 (44.1%)	< 0.001
	Received chemotherapy	12,430 (57.0%)	10,135 (57.4%)	927 (53.0%)	154 (64.7%)	1,214 (55.9%)	
Surgery	No/unknown	16,881 (77.4%)	13,464 (76.3%)	1,556 (89.0%)	189 (79.4%)	1,672 (77.0%)	< 0.001
	Surgery	4,933 (22.6%)	4,191 (23.7%)	193 (11.0%)	49 (20.6%)	500 (23.0%)	
Radiation	No/unknown	14,651 (67.2%)	11,805 (66.9%)	1,128 (64.5%)	159 (66.8%)	1,559 (71.8%)	< 0.001
	Received Radiation	7,163 (32.8%)	5,850 (33.1%)	621 (35.5%)	79 (33.2%)	613 (28.2%)	
Behavioral characteristic							
Smoking	Never	4,777 (21.9%)	3,673 (20.8%)	406 (23.2%)	73 (30.7%)	625 (28.8%)	< 0.001
	Current	4,568 (20.9%)	3,576 (20.3%)	541 (30.9%)	45 (18.9%)	406 (18.7%)	
	Former	7,579 (34.7%)	6,448 (36.5%)	446 (25.5%)	65 (27.3%)	620 (28.5%)	
	Unknown	4,890 (22.4%)	3,958 (22.4%)	356 (20.4%)	55 (23.1%)	521 (24.0%)	

*p*-values were calculated using Kruskal–Wallis tests for continuous variables and chi-square tests for categorical variables

*NH* non-Hispanic, *IQR* interquartile range

Histology was categorized as adenocarcinoma (8140–8147, 8255, 8260, 8310, 8323, 8480–8490, 8574), squamous cell carcinoma (8050–8084), and other/unspecified types

Esophageal cancer cases were identified using ICD-O-3 topography codes C15.0–C15.9

Stage is based on SEER Summary Stage (localized, regional, distant), which only approximately maps to American Joint Committee on Cancer (AJCC) Tumor-Node-Metastasis (TNM) system categories

We then fit multivariable Cox proportional hazards regression models to estimate adjusted hazard ratios (aHR) and 95% confidence intervals (CI) for mortality associated with race/ethnicity [22]. One all-patients (all stages combined) model was estimated for the full cohort (Model 1, Table 2), followed by three separate SEER Summary Stage-stratified models (localized, regional, distant; Models 2–4, Table 2). Because recommended therapies differ by stage, all stage-stratified Cox models included surgery, chemotherapy, and radiation as covariates [12–14]. Models were adjusted for age, sex, marital status, insurance type, neighborhood-level poverty, location, histologic subtype, tumor grade, treatment modalities (surgery, chemotherapy, radiation), and smoking at diagnosis (never, former, current, or unknown). The all-patients model was additionally adjusted for SEER Summary Stage; the stage-stratified models did not include stage as a covariate. Location and histology were entered as a single composite covariate (location–histology) using the categories above; upper was the reference group in all models.

**Table 2:** Adjusted Hazard Ratios for Esophageal Cancer-Specific Mortality Among Patients (all stages combined) and Stratified by Disease Stage, Florida Cancer Data System, 2005–2021

Variable	All Patients^†^ *n* = 21,756 aHR (95% CI)	Localized *n* = 4,152 aHR (95% CI)	Regional *n* = 6,366 aHR (95% CI)	Distant *n* = 6,507 aHR (95% CI)
Race/Ethnicity (ref: NH White)	Ref. (1.00)	Ref. (1.00)	Ref. (1.00)	Ref. (1.00)
NH Black	**1.08 (1.02–1.15)**	**1.24 (1.05–1.45)**	**1.19 (1.07–1.35)**	**0.88 (0.80–0.98)**
NH Asian/PI	1.13 (0.96–1.31)	0.81 (0.51–1.28)	**1.40 (1.07–1.83)**	1.10 (0.85–1.44)
Hispanic	0.95 (0.89–1.00)	1.09 (0.94–1.27)	0.98 (0.88–1.09)	**0.79 (0.72–0.86)**
Age Group (ref: 18–44)	Ref. (1.00)	Ref. (1.00)	Ref. (1.00)	Ref. (1.00)
45–54	1.09 (0.96–1.24)	1.19 (0.73–1.95)	1.13 (0.88–1.46)	1.16 (0.97–1.39)
55–64	**1.19 (1.05–1.34)**	1.24 (0.78–1.99)	**1.35 (1.07–1.72)**	1.13 (0.96–1.35)
65–74	**1.23 (1.08–1.39)**	1.41 (0.88–2.26)	**1.32 (1.03–1.69)**	1.10 (0.92–1.32)
75–84	**1.52 (1.33–1.73)**	**1.90 (1.18–3.06)**	**1.61 (1.25–2.07)**	**1.25 (1.04–1.51)**
85 +	**2.27 (1.98–2.60)**	**3.35 (2.06–5.44)**	**2.35 (1.79–3.09)**	**1.37 (1.11–1.70)**
Sex (ref: Female)	Ref. (1.00)	Ref. (1.00)	Ref. (1.00)	Ref. (1.00)
Male	**1.14 (1.09–1.19)**	1.05 (0.94–1.16)	**1.21 (1.12–1.31)**	**1.14 (1.06–1.23)**
Marital Status (ref: Married/Partner)	Ref. (1.00)	Ref. (1.00)	Ref. (1.00)	Ref. (1.00)
Unmarried	**1.22 (1.16–1.28)**	**1.24 (1.09–1.42)**	**1.17 (1.07–1.28)**	**1.17 (1.09–1.26)**
Separated/widowed	**1.16 (1.11–1.21)**	**1.26 (1.13–1.39)**	**1.15 (1.06–1.24)**	**1.10 (1.02–1.18)**
Unknown	1.00 (0.92–1.09)	1.04 (0.82–1.32)	1.17 (0.99–1.38)	**0.94 (0.81–1.10)**
Insurance (ref: Private)	Ref. (1.00)	Ref. (1.00)	Ref. (1.00)	Ref. (1.00)
Medicaid	**1.30 (1.21–1.40)**	**1.43 (1.14–1.80)**	**1.37 (1.19–1.58)**	**1.24 (1.12–1.38)**
Medicare	**1.05 (1.00–1.11)**	1.06 (0.92–1.23)	1.02 (0.92–1.12)	1.07 (0.99–1.16)
Uninsured	**1.32 (1.20–1.44)**	**1.66 (1.25–2.19)**	**1.25 (1.05–1.48)**	**1.26 (1.11–1.43)**
Other/Unknown	1.04 (0.99–1.12)	1.16 (0.97–1.39)	0.97 (0.86–1.09)	1.08 (0.98–1.20)
Neighborhood Poverty (ref: Lowest)	Ref. (1.00)	Ref. (1.00)	Ref. (1.00)	Ref. (1.00)
Low middle	1.03 (0.98–1.09)	0.96 (0.83–1.11)	**1.13 (1.02–1.25)**	1.02 (0.93–1.12)
High middle	**1.11 (1.04–1.16)**	1.03 (0.89–1.19)	**1.17 (1.06–1.29)**	**1.12 (1.02–1.23)**
Highest	**1.18 (1.11–1.25)**	1.16 (0.99–1.36)	**1.32 (1.18–1.48)**	1.10 (1.00–1.22)
Stage (ref: Localized)	Ref. (1.00)	Ref. (1.00)	-	-
Regional	**1.83 (1.74–1.94)**	-	Ref. (1.00)	-
Distant	**3.36 (3.18–3.55)**	-	-	Ref. (1.00)
Unknown	**1.23 (1.16–1.31)**	-	-	-
Grade (ref: Well differentiated (grade 1))	Ref. (1.00)	Ref. (1.00)	Ref. (1.00)	Ref. (1.00)
Moderately differentiated (grade 2)	**1.19 (1.08–1.30)**	1.14 (0.95–1.36)	**1.25 (1.06–1.48)**	**1.26 (1.03–1.53)**
Poorly differentiated (grade 3)	**1.45 (1.32–1.59)**	**1.52 (1.26–1.83)**	**1.55 (1.31–1.83)**	**1.52 (1.25–1.84)**
Undifferentiated (grade 4)	**1.50 (1.23–1.83)**	0.96 (0.47–1.96)	1.43 (0.99–2.06)	**1.82 (1.34–2.46)**
Unknown	1.01 (0.93–1.11)	0.92 (0.77–1.10)	1.17 (0.99–1.38)	**1.27 (1.05–1.53)**
Location and Histology (ref: Upper)	Ref. (1.00)	Ref. (1.00)	Ref. (1.00)	Ref. (1.00)
Lower adenocarcinoma	**1.08 (1.01–1.16)**	**1.21 (1.03–1.46)**	**1.23 (1.10–1.39)**	0.93 (0.82–1.05)
Lower non-adenocarcinoma	**1.14 (1.06–1.23)**	**1.35 (1.11–1.65)**	**1.24 (1.08–1.42)**	1.07 (0.94–1.23)
Middle	**1.11 (1.03–1.19)**	**1.34 (1.11–1.62)**	**1.18 (1.04–1.35)**	0.94 (0.83–1.08)
Overlapping/others	**1.20 (1.11–1.29)**	**1.61 (1.32–1.94)**	**1.33 (1.15–1.53)**	1.00 (0.88–1.14)
Chemotherapy (ref: Received)	Ref. (1.00)	Ref. (1.00)	Ref. (1.00)	Ref. (1.00)
Not received /unknown	**1.71 (1.64–1.77)**	1.03 (0.93–1.15)	**1.73 (1.60–1.88)**	**2.99 (2.82–3.18)**
Surgery (ref: Received)	Ref. (1.00)	Ref. (1.00)	Ref. (1.00)	Ref. (1.00)
Not received /unknown	**2.51 (2.39–2.63)**	**3.45 (3.09–3.86)**	**2.01 (1.88–2.16)**	**2.13 (1.88–2.42)**
Radiation (ref: Received)	Ref. (1.00)	Ref. (1.00)	Ref. (1.00)	Ref. (1.00)
Not received /unknown	**1.12 (1.08–1.17)**	**1.22 (1.09–1.37)**	**1.12 (1.04–1.21)**	**1.23 (1.16–1.31)**
Tobacco (ref: Never smoker)	Ref. (1.00)	Ref. (1.00)	Ref. (1.00)	Ref. (1.00)
Current	**1.14 (1.08–1.19)**	**1.37 (1.20–1.57)**	**1.16 (1.06–1.28)**	1.01 (0.93–1.09)
Former	0.96 (0.92–1.01)	1.05 (0.94–1.18)	1.02 (0.94–1.11)	0.94 (0.88–1.01)
Unknown	0.95 (0.91–1.00)	1.00 (0.88–1.15)	1.03 (0.94–1.14)	**0.82 (0.76–0.90)**

Disease stage was categorized using the Surveillance, Epidemiology, and End Results (SEER) Summary Stage classification (localized, regional, distant), which approximately maps to American Joint Committee on Cancer (AJCC) Tumor-Node-Metastasis (TNM) staging system

Esophageal cancer cases were identified using ICD-O-3 topography codes C15.0–C15.9

Because disease stage is the stratifying variable in Models 2–4; stage coefficients are only shown in the all-patients model (Model 1)

All-patients model additionally adjusted for SEER Summary Stage; stage-stratified models did not include stage as a covariate

Bolded estimates indicate statistical significance at *p* < 0.05

*CI* confidence interval; *aHR* adjusted hazard ratio, *PI* pacific Islanders

To assess the robustness of our findings, we conducted several sensitivity analyses. First, we re-estimated models including racial/ethnic groups with smaller sample sizes (e.g., American Indian/Alaska Native, “Other”) and patients with nonbinary or other sex classifications that were excluded from the main analysis for model stability; these results are presented in Supplementary Table 2S. Second, because recommended therapies differ by SEER Summary Stage, we conducted guideline-concordant, stage-specific sensitivity analyses [12–14]. For localized disease, we re-fit the Cox model including surgery and radiation; for regional disease, we retained surgery, chemotherapy, and radiation; and for distant disease, we included chemotherapy and radiation (Supplementary Table 3S).

In addition to survival models, we used multivariable logistic regression to assess racial and ethnic differences in the receipt of treatment (surgery, chemotherapy, radiation) (Table 3). These models were adjusted for age, sex, marital status, insurance type, neighborhood poverty, stage, histology, grade, tumor location, and smoking status. Because treatment indications differ by stage, we additionally estimated stage-specific logistic regression models for localized, regional, and distant disease (Supplementary Table 4S).

**Table 3:** Adjusted Odds Ratios (AOR) for Receipt of Chemotherapy, Surgery, or Radiation by Race/Ethnicity Among Esophageal Cancer Patients, Florida Cancer Data System, 2005–2021 (*n* = 21,814)

Race/Ethnicity	Model 1: Chemotherapy AOR (95% CI)	Model 2: Surgery AOR (95% CI)	Model 3: Radiation AOR (95% CI)
NH White (Ref)	Ref	Ref	Ref
NH Black	**0.79 (0.70–0.90)**	**0.51 (0.43–0.61)**	1.14 (0.99–1.30)
NH Asian/Pacific Islanders	1.31 (0.95–1.80)	0.86 (0.60–1.23)	0.89 (0.64–1.23)
Hispanic	1.04 (0.93–1.16)	1.11 (0.98–1.26)	**0.78 (0.69–0.88)**

All models are adjusted for age group, sex, marital status, insurance type, neighborhood poverty, summary stage, histologic type, tumor grade, tumor location, smoking status, and additionally:

Model 1 (Chemotherapy): adjusted for surgery and radiation

Model 2 (Surgery**)**: adjusted for chemotherapy and radiation

Model 3 (Radiation)**:** adjusted for chemotherapy and surgery

*AOR* adjusted odds ratio, *CI* confidence interval, *NH* non-Hispanic

Neighborhood poverty level was derived from U.S. Census tract-based indicators at the time of diagnosis

*p*-values < 0.05 are bolded

Proportional hazards assumptions were assessed using Schoenfeld residuals, and multicollinearity was evaluated using variance inflation factors (mean VIF = 2.72 for Cox models; approximately 3.0 for logistic regression models) [23]. Goodness-of-fit statistics and residual plots were also used to evaluate model performance [24]. Model fit for logistic regression models was additionally assessed using the Hosmer–Lemeshow goodness-of-fit test [25]. All analyses were conducted using Stata 18.0 (StataCorp, College Station, TX). This study was deemed exempt by the University of Miami Institutional Review Board and the Florida Department of Health, as it used de-identified registry data.

## Results

### Patient characteristics

Among 21,814 patients, most were NH White (81.0%), followed by NH Black (8.0%), Hispanic (10.0%) and NH Asian (1.1%). Compared to NH White patients, NH Black patients were younger, more often unmarried, uninsured, or enrolled in Medicaid and were more likely to reside in high-poverty neighborhoods. They also had a substantially higher prevalence of squamous cell carcinoma (71.8 vs 22.9%) and lower use of surgery than NH White patients (11.0 vs 23.7%) (*p* < 0.01) (Table 1).

### Cancer-specific survival by race/ethnicity

Figure 2 shows 5-year Kaplan–Meier survival curves for cancer-specific survival by race and ethnicity. Survival differed significantly across groups (log-rank *p* < 0.001), with NH Black patients showing the lowest survival and NH White patients the highest. Hispanic and NH Asian/Pacific Islander patients had intermediate survival probabilities. Across all groups, the largest decline in survival occurred during the first year after diagnosis.

### Multivariable survival analyses

Table 2 presents Cox proportional hazards model results. In the full cohort, NH Black patients had a significantly higher risk of death compared to NH White patients (adjusted hazard ratio [aHR]: 1.08; 95% CI 1.02–1.15). In stage-stratified models, NH Black patients had elevated mortality risk at the localized (Model 2: aHR: 1.24; 95% CI 1.05–1.45) and regional stages (Model 3: aHR: 1.19; 95% CI 1.07–1.35), but lower mortality at the distant stage (Model 4: aHR: 0.88; 95% CI 0.80–0.98). Hispanic patients had similar mortality to NH White patients at the localized and regional stages and lower mortality at the distant stage (aHR: 0.79; 95% CI 0.72–0.86).

Other predictors of mortality were consistent across models (Table 2). Older age was associated with higher mortality risk, particularly among patients aged 85 + vs. those aged 18–44 years (aHR: 2.27; 95% CI 1.98–2.60). Male sex vs. female, higher tumor grade, and advanced tumor stage were also linked to worse survival (Model 1, Table 2). Tumor location–histology mattered, as overlapping/other was associated with higher mortality relative to upper esophagus (aHR: 1.20; 95% CI 1.11–1.29).

Socioeconomic and behavioral factors also influenced outcomes. Compared to private insurance, patients with Medicaid (aHR: 1.30; 95% CI 1.21–1.40), Medicare (aHR: 1.05, 95% CI 1.00–1.11), or no insurance (aHR: 1.32; 95% CI 1.20–1.44) had a higher risk of death (Model 1, Table 2). Living in the highest poverty quartiles vs. the lowest (aHR: 1.18; 95% CI 1.11–1.25) and current smoking vs. never smoking (aHR: 1.14; 95% CI 1.08–1.19) were both associated with elevated mortality risk. Former smoking was not significantly different from never smoking, indicating that continued tobacco exposure adversely affects survival. Being married or living with a partner was protective relative to unmarried patients across all models (Table 2).

### Stage-specific treatment and survival

Stratified analyses by stage (Table 1S) highlighted expected shifts in treatment patterns, including higher surgery use in localized disease and greater multimodality therapy in regional disease. Because recommended therapies differ by disease stage, these treatment associations are interpreted within this stage-specific clinical framework [12–14]. In the all-patients (all stages combined) model, not receiving surgery (aHR: 2.51; 95% CI 2.39–2.63), chemotherapy (aHR: 1.71; 95% CI 1.64–1.77), or radiation (aHR: 1.12; 95% CI 1.08–1.17) was strongly associated with significantly higher mortality (Model 1, Table 2). Stage-specific analyses revealed patterns consistent with guideline-based care.

Localized stage: Not receiving surgery was most strongly associated with mortality (Model 2: aHR: 3.45; 95% CI 3.09–3.86).

Regional stage: Not receiving chemotherapy had the largest impact (Model 3: aHR 1.73; 95% CI 1.60–1.88).

Distant stage: Chemotherapy was again the dominant predictor (Model 4: aHR: 2.99; 95% CI 2.82–3.18).

Radiation therapy: Not receiving radiation therapy was also associated with higher mortality across all stages (localized: aHR 1.22, 95% CI 1.09–1.37; regional: aHR 1.12, 95% CI 1.04–1.21; distant: aHR 1.23, 95% CI 1.16–1.31). However, the magnitude of these associations was smaller than those of surgery in the localized stage and chemotherapy in the regional and distant stages, consistent with radiation’s role as part of multimodality therapy or as a palliative measure rather than the primary curative modality (Models 2–4, Table 2).

Sensitivity analyses including models with all racial/ethnic groups and sex categories (Supplementary Table 2S) and models restricting covariates to stage-appropriate therapies (Supplementary Table 3S) yielded consistent results, reinforcing surgery as the key predictor in localized disease and chemotherapy in regional and distant disease.

### Treatment disparities

Table 3 presents overall logistic regression models of treatment receipt. NH Black patients had significantly lower odds of undergoing surgery (adjusted odds ratio [aOR]: 0.51; 95% CI 0.43–0.61) and chemotherapy (aOR: 0.79; 95% CI 0.70–0.90) compared with NH White patients. Hispanic patients had lower odds of receiving radiation therapy (aOR: 0.78; 95% CI 0.69–0.88) but did not differ significantly in surgery or chemotherapy receipt.

When stratified by SEER Summary Stage, disparities persisted and were most pronounced in localized disease. NH Black patients had markedly lower odds of receiving surgery in both localized (aOR: 0.38; 95% CI 0.27–0.55) and regional disease (aOR: 0.63; 95% CI 0.49–0.81). Chemotherapy disparities were also evident for regional (aOR: 0.69; 95% CI 0.53–0.89) and distant disease (aOR: 0.79; 95% CI 0.64–0.97). Full stage-specific estimates are provided in Supplemental Table 4S.

## Discussion

In this population-based analysis of esophageal cancer patients in Florida, we found that NH Black patients experienced significantly higher mortality than NH White patients, particularly at localized and regional stages, when treatment is most likely to be curative. These disparities persisted after adjustment for demographic, clinical, and socioeconomic factors. In contrast, we did not observe a survival disadvantage at the distant stage, where uniformly poor prognosis may attenuate the impact of differential access to care and treatment intensity. This pattern may also reflect differences in treatment selection, competing risks, or cause-of-death misclassification at advanced stages.

These findings are consistent with prior national SEER-based analyses showing that NH Black patients with localized esophageal squamous cell carcinoma experience markedly lower survival than NH White patients, even after adjustment for treatment and clinical variables [3]. In contrast to SEER–Medicare analyses, which found early-stage racial/ethnic gaps were largely explained by lower surgery use, our adjusted models still showed residual disparities, suggesting additional structural factors in Florida [4, 18, 26]. Florida may represent a particularly important context for these disparities, as it is a Medicaid non-expansion state with substantial heterogeneity in access to specialty oncology care [27, 28]. Prior work using FCDS data has also demonstrated significant disparities in cancer outcomes associated with socioeconomic status and geographic variation across the state [29]. In such settings, underinsurance and financial barriers to care, delays in diagnostic workup, transportation barriers, fragmented referral pathways, and differential access to high-volume surgical centers may disproportionately affect historically marginalized populations and contribute to worse outcomes, even after adjustment for insurance type and neighborhood poverty [27–31].

We further show that NH Black patients were less likely to undergo surgery and chemotherapy, even after adjusting for insurance type and neighborhood poverty, consistent with prior reports of inequities in cancer care, including insurance-related barriers to treatment [3, 32–36]. These differences likely reflect multiple structural and healthcare system-level barriers to care. In particular, differential access to high-volume surgical centers and specialist referral networks may limit receipt of curative therapies, while delays in diagnostic workup and care coordination challenges may further reduce timely treatment initiation. In addition, patient–provider communication and other barriers to engagement with care may influence care-seeking behavior, treatment decision-making, and treatment acceptance [30, 37–39]. These treatment gaps likely mediate survival differences, particularly at curative stages.

Another finding that warrants further explanation is why Hispanic patients had lower odds of radiation therapy compared with NH White patients. Potential explanations, including differences in access to radiation facilities, care coordination, and cultural or perceptual factors related to treatment modalities, warrant further investigation.

The concentration of disparities at curative stages underscores missed opportunities for early intervention. Residual inequities after adjustment for insurance and poverty suggest deeper systemic barriers, including healthcare navigation challenges such as difficulties coordinating referrals, accessing specialty care, and managing insurance-related complexity, as well as institutional mistrust shaped by historical and ongoing inequities in healthcare delivery [17]. Cumulative disadvantage theory posits that small, repeated inequities across the care continuum, from diagnosis through treatment, can accumulate to produce population-level disparities in outcomes [40].

NH Black patients also had a higher prevalence of squamous cell carcinoma, a histologic subtype linked to modifiable behavioral risk factors like smoking and alcohol use that are, in turn, shaped by structural disadvantage [41, 42]. NH Black patients were additionally more likely to reside in high-poverty neighborhoods, be uninsured or covered by Medicaid, and present with advanced-stage disease, factors that may contribute to poorer survival outcomes [43, 44].

Despite lower treatment rates, Hispanic patients did not exhibit significantly worse survival, consistent with the “Hispanic paradox,” whereby Hispanic populations have comparable or better outcomes despite socioeconomic disadvantage [45]. Proposed explanations include selective migration (“healthy migrant” effect), return migration at the end of life (“salmon bias”), and stronger social and family support networks that may influence health behaviors and engagement with care [45, 46]. These mechanisms warrant further investigation.

Current smoking was independently associated with higher mortality, whereas former smoking was not, indicating that ongoing tobacco use at diagnosis worsens prognosis. This provides the first population-based confirmation using statewide registry data that smoking at diagnosis independently predicts worse survival in esophageal cancer, consistent with prior surgical and radiotherapy studies [10, 11].

This study has limitations. First, misclassification of cause-of-death is possible despite our focus on cancer-specific mortality. Second, the registry lacks data on comorbidities, performance status, provider characteristics, and patient preferences, which may influence treatment and outcomes. Third, we could not assess timeliness or quality of surgical and nursing care. Fourth, we relied on neighborhood-level rather than individual-level socioeconomic measures. Fifth, because this analysis was conducted in a single state, findings may not be generalizable to the broader U.S. population, particularly states with different insurance environments, healthcare infrastructure, and racial/ethnic composition. Sixth, approximately one-fifth of patients had missing smoking status, which may have introduced residual confounding. We retained these individuals using an “unknown” category to preserve the population-based nature of the study and avoid excluding a substantial portion of the cohort, though residual confounding from unmeasured smoking behavior cannot be fully ruled out. Finally, migration at the end of life, particularly among foreign-born individuals who may return to their countries of origin, may lead to undercount of mortality (“salmon bias”) and bias survival estimates [47].

Further research should clarify drivers of these disparities, including qualitative studies (e.g., interviews and focus groups) examining patient–provider interactions, care experiences, and barriers to treatment among NH Black communities [48]. Health services research investigating referral networks, provider availability, and hospital quality could help identify modifiable system-level contributors. Finally, policy evaluations, particularly on the potential impact of Medicaid expansion in Florida, may help shape equitable solutions.

In conclusion, this study highlights persistent, stage-specific racial and ethnic disparities in esophageal cancer care and survival outcomes in Florida. NH Black patients, especially those diagnosed at earlier stages, experience lower treatment rates and worse outcomes compared to their NH White counterparts. These findings highlight the need for structural and system-level interventions to ensure equitable care delivery at stages where survival is most modifiable. Addressing these disparities requires a multifaceted approach such as timely diagnosis, equitable referral and treatment, enhanced cultural competence among providers, and systemic reforms targeting structural determinants of health.

## Supplementary Information

Below is the link to the electronic supplementary material.

Supplementary file1 (DOCX 30 KB)

## Data Availability

No datasets were generated or analyzed during the current study.
