# Supplementary material for: Racial and ethnic disparities in esophageal cancer survival are greatest at curable stages: a population-based study
Source: Cancer Causes Control. 2026 Jun 30;37(7):115. doi: 10.1007/s10552-026-02194-5 (PMC13320043; doi:10.1007/s10552-026-02194-5)
Supplement: Supplementary file 1 — Supplementary file1 (DOCX 30 KB) [file 10552_2026_2194_MOESM1_ESM.docx]

**Supplementary Tables**

| **Table S1. Characteristics of Esophageal Cancer Patients by Disease Stage, Florida Cancer Data System, 2005–2021** | | | | | | |
| --- | --- | --- | --- | --- | --- | --- |
| Variable | Subcategory | **All Patients** (N=21,814) n (%) | **Localized**  (n=4,160)  n (%) | **Regional**  (n=6,370)  n (%) | **Distant** (n=6,514)  n (%) | p-value |
| **Demographics** |  |  |  |  |  |  |
| Age, median (IQR), years |  | 69 (61–77) | 71 (64–79) | 68 (61–76) | 67 (59–75) | <0.001 |
| Age Group | 18–44 | 410 (1.9%) | 42 (1.0%) | 127 (2.0%) | 171 (2.6%) | <0.001 |
|  | 45–54 | 1,892 (8.7%) | 255 (6.1%) | 592 (9.3%) | 753 (11.6%) |  |
|  | 55–64 | 5,159 (23.6%) | 822 (19.8%) | 1,651 (25.9%) | 1,835 (28.2%) |  |
|  | 65–74 | 7,191 (33.0%) | 1,414 (34.0%) | 2,171 (34.1%) | 2,097 (32.2%) |  |
|  | 75–84 | 5,383 (24.7%) | 1,197 (28.8%) | 1,490 (23.4%) | 1305 (20.0%) |  |
|  | 85+ | 1,779 (8.2%) | 430 (10.3%) | 339 (5.3%) | 353 (5.4%) |  |
| Sex | Male | 17,092 (78.4%) | 3,206 (77.1%) | 4,973 (78.1%) | 5,339 (82.0%) | <0.001 |
|  | Female | 4,722 (21.6%) | 954 (22.9%) | 1,397 (21.9%) | 1,175 (18.0%) |  |
| Race/Ethnicity | NH White | 17,655 (80.9%) | 3,439 (82.7%) | 5,127 (80.5%) | 5,237 (80.4%) | 0.153 |
|  | NH Black | 1,749 (8.0) | 304 (7.3%) | 515 (8.1%) | 551 (8.5%) |  |
|  | NH Asian/Pacific Islander | 238 (1.1) | 38 (0.9%) | 81 (1.3%) | 68 (1.0%) |  |
|  | Hispanic | 2,172 (10.0) | 379 (9.1%) | 647 (10.2%) | 658 (10.1%) |  |
| Marital Status | Unmarried | 3,675 (16.8%) | 595 (11.9%) | 1073 (14.1%) | 1267 (16.2%) | <0.001 |
|  | Married or living with a partner | 12,022 (55.1%) | 2483 (49.7%) | 3721 (49.0%) | 3592 (46.0%) |  |
|  | Separated/widowed | 4,861 (22.3%) | 930 (18.6%) | 1,347 (17.7%) | 1,433 (18.3%) |  |
|  | Unknown | 1,256 (5.8%) | 152 (3.0%) | 229 (3.0%) | 222 (2.8%) |  |
| Insurance | Private | 4,442 (20.4%) | 785 (18.9%) | 1,482 (23.3%) | 1,539 (23.6%) | <0.001 |
|  | Medicaid | 1,276 (5.8%) | 165 (4.0%) | 351 (5.5%) | 561 (8.6%) |  |
|  | Medicare | 12,542 (57.5%) | 2,706 (65.0%) | 3,637 (57.1%) | 3,410 (52.3%) |  |
|  | Uninsured | 766 (3.5%) | 102 (2.5%) | 216 (3.4%) | 333 (5.1%) |  |
|  | Other | 2,788 (12.8%) | 402 (9.7%) | 684 (10.7%) | 671 (10.3%) |  |
| Poverty Level | Lowest | 2,613 (12.0%) | 509 (9.9%) | 824 (10.6%) | 742 (9.3%) | <0.001 |
|  | Low-middle | 6,520 (29.9%) | 1,257 (24.5%) | 1,923 (24.7%) | 1,927 (24.1%) |  |
|  | High-middle | 8,041 (36.9%) | 1,544 (30.1%) | 2,333 (29.9%) | 2,377 (29.8%) |  |
|  | Highest | 4,461 (20.5%) | 828 (16.1%) | 1,252 (16.1%) | 1,417 (17.7%) |  |
|  | Unknown | 179 (0.8%) | 22 (0.4%) | 38 (0.5%) | 51 (0.6%) |  |
| **Tumor Characteristics** |  |  |  |  |  |  |
| Stage | Localized | 4,160 (19.1%) | - | - | - | - |
|  | Regional | 6,370 (29.2%) | - | - | - |  |
|  | Distant | 6,514 (29.9%) | - | - | - |  |
|  | Unknown | 4,770 (21.9%) | - | - | - |  |
| Histology | Adenocarcinoma | 13,054 (59.8%) | 2,759 (66.3%) | 4,057 (63.7%) | 4,202 (64.5%) | <0.001 |
|  | Squamous cell carcinoma | 6,161 (28.2%) | 1,175 (28.2%) | 2046 (32.1%) | 1,629 (25.0%) |  |
|  | Other | 2,599 (11.9%) | 226 (5.4%) | 267 (4.2%) | 683 (10.5%) |  |
| Grade | Well differentiated (grade 1) | 797 (3.7%) | 317 (7.6%) | 226 (3.5%) | 130 (2.0%) | <0.001 |
|  | Moderately differentiated (grade 2) | 4,412 (20.2%) | 1,041 (25.0%) | 1,533 (24.1%) | 1,217 (18.7%) |  |
|  | Poorly differentiated  (grade 3) | 5,381 (24.7%) | 776 (18.7%) | 1,802 (28.3%) | 2,065 (31.7%) |  |
|  | Undifferentiated  (grade 4) | 159 (0.7%) | 20 (0.5%) | 48 (0.8%) | 76 (1.2%) |  |
|  | Unknown | 11,065 (50.7%) | 2,006 (48.2%) | 2,761 (43.3%) | 3,026 (46.5%) |  |
| Tumor Location | Middle/lower third | 15,447 (70.8%) | 3,175 (76.3%) | 4,990 (78.3%) | 4,704 (72.2%) | <0.001 |
|  | Overlapping | 770 (3.5%) | 90 (2.2%) | 220 (3.5%) | 329 (5.1%) |  |
|  | Other | 5,597 (25.7%) | 895 (21.5%) | 1,160 (18.2%) | 1,481 (22.7%) |  |
| **Treatment characteristics** |  |  |  |  |  |  |
| Chemotherapy | No/unknown | 9,384 (43.0%) | 2,531 (60.8%) | 1,314 (20.6%) | 2,448 (37.6%) | <0.001 |
|  | Received chemotherapy | 12,430 (57.0%) | 1,629 (39.2%) | 5,056 (79.4%) | 4,066 (62.4%) |  |
| Surgery | No Surgery | 16,881 (77.4%) | 2,409 (57.9%) | 4,100 (64.4%) | 6,162 (94.6%) | <0.001 |
|  | Surgery | 4,933 (22.6%) | 1,751 (42.9%) | 2,270 (35.6%) | 352 (5.4%) |  |
| Radiation | No/unknown | 14,651 (67.2%) | 3,051 (73.3%) | 3,193 (50.1%) | 4,545 (69.8%) | <0.001 |
|  | Received Radiation | 7,163 (32.8%) | 1,109 (26.7%) | 3,177 (49.9%) | 1,969 (30.2%) |  |
| **Behavioral characteristic** |  |  |  |  |  |  |
| Smoking | Never | 4,777 (21.9%) | 1,023 (23.6%) | 1,352 (20.4%) | 1,398 (20.6%) | <0.001 |
|  | Current | 4,568 (20.9%) | 761 (17.6%) | 1,525 (23.0%) | 1,546 (22.8%) |  |
|  | Former | 7,579 (34.7%) | 1,557 (35.9%) | 2,302 (34.7%) | 2,321 (34.2%) |  |
|  | Unknown | 4,890 (22.4%) | 819 (18.9%) | 1,191 (17.9%) | 1,249 (18.4%) |  |
| Notes:   - P-values were calculated using Kruskal–Wallis tests for continuous variables and chi-square tests for categorical variables. - Stage is based on SEER Summary Stage (localized, regional, distant), which only approximately maps to American Joint Committee on Cancer (AJCC) Tumor-Node-Metastasis (TNM) system categories. - Tumor histology was grouped as: Adenocarcinoma (ICD-O-3 codes 8140–8147, 8255, 8260, 8310, 8323, 8480–8490, 8574), Squamous Cell Carcinoma (8050–8084), and Other/Unspecified. - Esophageal cancer cases were identified using ICD-O-3 Topography codes C15.0–C15.9. - Neighborhood poverty level was derived from U.S. Census tract-based indicators at the time of diagnosis. | | | | | | |

| **Table S2. Sensitivity Analysis: Adjusted Hazard Ratios for Mortality Among Esophageal Cancer Patients, Including All Sex and Racial Groups, (all-stages combined) and Stratified by Disease Stage, Florida Cancer Data System, 2005–2021** | | | | |
| --- | --- | --- | --- | --- |
| **Race/Ethnicity** | **All Patients** (Model 1)  aHR (95% CI) | **Localized**  (Model 2)  aHR (95% CI) | **Regional**  (Model 3)  aHR (95% CI) | **Distant**  (Model 4)  aHR (95% CI) |
| NH White (ref) | Ref. 1.00 | Ref. 1.00 | Ref. 1.00 | Ref. 1.00 |
| NH Black | **1.08 (1.01–1.15)** | **1.21 (1.04–1.44)** | **1.20 (1.07–1.35)** | **0.88 (0.80–0.98)** |
| NH Asian/PI | 1.12 (0.96–1.31) | 0.78 (0.47–1.17) | **1.40 (1.07–1.83)** | 1.10 (0.85–1.44) |
| Hispanic | **0.94 (0.89–1.00)** | 1.07 (0.92–1.24) | 0.98 (0.88–1.09) | **0.79 (0.72–0.87)** |
| Others | **0.57 (0.42–0.77)** | 0.80 (0.27–1.95) | 0.72 (0.41–1.23) | **0.40 (0.24–0.65)** |
| Notes:   - Adjusted Cox models control for age, sex (including “Other”), insurance, poverty, marital status, tumor stage, location, histology, grade, treatment modalities (surgery, chemotherapy, radiation), and smoking status. - This analysis includes racial/ethnic groups (e.g., American Indian/Alaska Native, Unknown) and sex categories that were excluded in the main analysis for model stability. - Stage is based on SEER Summary Stage (localized, regional, distant), which only approximately maps to American Joint Committee on Cancer (AJCC) Tumor-Node-Metastasis (TNM) system categories. - P-values < 0.05 are bolded. - Abbreviations: aHR: Adjusted Hazard Ratio; CI: Confidence Interval | | | | |

| **Table S3. Sensitivity Analyses: Stage-Specific Cox Proportional Hazards Models by Guideline-Concordant Treatment, Florida Cancer Data System, 2005–2021** | | | |
| --- | --- | --- | --- |
| **Race/Ethnicity** | **Localized**  (Model 2)  aHR (95% CI)  n=4,152 | **Regional**  (Model 3)  aHR (95% CI)  n=6,366 | **Distant**  (Model 4)  aHR (95% CI)  n=6,507 |
| NH White (ref) | Ref. 1.00 | Ref. 1.00 | Ref. 1.00 |
| NH Black | **1.24 (1.05–1.45)** | **1.20 (1.07–1.35)** | **0.89 (0.81–0.99)** |
| NH Asian/PI | 0.80 (0.51–1.27) | **1.40 (1.07–1.83)** | 1.07 (0.83–1.40) |
| Hispanic | 1.09 (0.94–1.27) | 0.98 (0.88–1.09) | **0.78 (0.71–0.85)** |
| **Notes:**   - Stage-specific Cox proportional hazards models were re-fit to align with guideline-concordant treatment definitions. • Localized disease: models included **surgery and radiation**. • Regional disease: models included **surgery, chemotherapy, and radiation.** • Distant disease: models included **chemotherapy and radiation**. - Stage is based on SEER Summary Stage (localized, regional, distant), which only approximately maps to American Joint Committee on Cancer (AJCC) Tumor-Node-Metastasis (TNM) system categories. - All models additionally adjusted for age, sex, insurance type, neighborhood poverty, marital status, tumor location, histology, grade, and smoking status. - Statistical significance was defined as p < 0.05 (significant values shown in **bold**). - **Abbreviations:** aHR = adjusted hazard ratio; CI = confidence interval. | | | |

| **Table S4. Stage-Specific Adjusted Odds Ratios (AOR) for Receipt of Surgery and Chemotherapy by Race/Ethnicity, Florida Cancer Data System, 2005–2021** | | | | | |
| --- | --- | --- | --- | --- | --- |
|  | **Surgery AOR** | | **Chemotherapy AOR** | | |
| **Race/Ethnicity** | **Localized**  (Model 1)  aOR (95% CI)  n=4,160 | **Regional**  (Model 2)  aOR (95% CI)  n=6,370 | **Localized**  (Model 3)  aOR (95% CI)  n=4,160 | **Regional**  (Model 4)  aOR (95% CI)  n=6,370 | **Distant**  (Model 5)  aOR (95% CI)  n=6,514 |
| NH White (ref) | Ref. 1.00 | Ref. 1.00 | Ref. 1.00 | Ref. 1.00 | Ref. 1.00 |
| NH Black | **0.38 (0.27–0.55)** | **0.63 (0.49–0.81)** | 0.98 (0.71–1.36) | **0.69 (0.53–0.89)** | **0.79 (0.64**–**0.97)** |
| NH Asian/PI | **0.36 (0.16–0.78)** | 1.05 (0.63–1.73) | 1.58 (0.75–3.31) | 1.23 (0.65–2.33) | 1.27 (0.72–2.26) |
| Hispanic | 0.90 (0.70-1.16) | 1.26 (1.04–1.52) | 1.14 (0.87–1.50) | 1.21 (0.95–1.54) | 0.99 (0.83–1.20) |
| **Notes:**   - **Models adjusted for age, sex, marital status, insurance, neighborhood poverty, tumor histology, tumor location, tumor grade, and smoking.** - Stage is based on SEER Summary Stage (localized, regional, distant), which only approximately maps to American Joint Committee on Cancer (AJCC) Tumor-Node-Metastasis (TNM) system categories. - **Surgery models include only localized and regional stages because surgery is not a standard treatment in distant disease. Chemotherapy** models are additionally adjusted for surgery and radiation. - **Chemotherapy models include all three stages (localized, regional, distant).** - **Statistical significance defined as p < 0.05 (bold in final manuscript).** - **Abbreviations: AOR = Adjusted Odds Ratio; CI = Confidence Interval; NH = Non-Hispanic.** | | | | | |

ss
